# Postnatal isoform switch and protein localization of LEF1 and TCF7L2 transcription factors in cortical, thalamic, and mesencephalic regions of the adult mouse brain

**DOI:** 10.1007/s00429-012-0474-6

**Published:** 2012-11-15

**Authors:** A. Nagalski, M. Irimia, L. Szewczyk, J. L. Ferran, K. Misztal, J. Kuznicki, M. B. Wisniewska

**Affiliations:** 1 Laboratory of Neurodegeneration, International Institute of Molecular and Cell Biology, 4 Ks. Trojdena Street, 02-109 Warsaw, Poland; 2 Banting and Best Department of Medical Research, Donnelly Centre, University of Toronto, Toronto, ON M5S 3E1 Canada; 3 Department of Human Anatomy and Psychobiology, School of Medicine, University of Murcia, Murcia, E30071 Spain; 4 Department of Molecular and Cellular Neurobiology, Nencki Institute of Experimental Biology, 3 Pasteur Street, 02-093 Warsaw, Poland

**Keywords:** β-Catenin, LEF1, TCF7L2, Alternative splicing, Brain, Thalamus

## Abstract

β-Catenin signaling, leading to the activation of lymphoid enhancer-binding factor 1/T cell factor (LEF1/TCF) transcription factors, plays a well-established role in transcription regulation during development and tissue homeostasis. In the adult organism, the activity of this pathway has been found in stem cell niches and postmitotic thalamic neurons. Recently, studies show that mutations in components of β-catenin signaling networks have been associated with several psychiatric disorders, indicating the involvement of β-catenin and LEF1/TCF proteins in the proper functioning of the brain. Here, we report a comprehensive analysis of LEF1/TCF protein localization and the expression profile of their isoforms in cortical, thalamic, and midbrain regions in mice. We detected LEF1 and TCF7L2 proteins in neurons of the thalamus and dorsal midbrain, i.e., subcortical regions specialized in the integration of diverse sources of sensory information. These neurons also exhibited nuclear localization of β-catenin, suggesting the involvement of β-catenin/LEF1/TCF7L2 in the regulation of gene expression in these regions. Analysis of alternative splicing and promoter usage identified brain-specific TCF7L2 isoforms and revealed a developmentally coordinated transition in the composition of LEF1 and TCF7L2 isoforms. In the case of TCF7L2, the typical brain isoforms lack the so-called C clamp; in addition, the dominant-negative isoforms are predominant in the embryonic thalamus but disappear postnatally. The present study provides a necessary framework to understand the role of LEF1/TCF factors in thalamic and midbrain development until adulthood and predicts that the regulatory role of these proteins in the adult brain is significantly different from their role in the embryonic brain or other non-neural tissues.

## Introduction

Lymphoid enhancer-binding factor 1/T cell factors (LEF1/TCFs) are transcription factors activated by β-catenin in the canonical Wnt signaling pathway. The induction of this pathway, also called the Wnt/β-catenin pathway, leads to the inhibition of β-catenin degradation and its subsequent accumulation in the cytoplasm. β-Catenin can then be translocated to the nucleus, bind to LEF1/TCF proteins, and activate its target genes (Angers and Moon 2009; MacDonald et al. 2009; Archbold et al. 2012). Canonical Wnt signaling controls fundamental aspects of animal development, such as the establishment of embryonic body plan, the expansion of progenitor cells, and lineage decisions in every major organ, including the central nervous system (McMahon et al. 1992; Galceran et al. 2000; Mukhopadhyay et al. 2001; Maretto et al. 2003; Logan and Nusse 2004; Zhou et al. 2004; Ciani and Salinas 2005; Grigoryan et al. 2008; Petersen and Reddien 2009; Freese et al. 2010; Niehrs 2010). In the adult, Wnt signaling activity is largely restricted to stem cell reservoirs (Maretto et al. 2003; Haegebarth and Clevers 2009; Wend et al. 2010). For example, Wnt signaling has been reported in neural progenitor cells in the subgranular zone of the hippocampus (Lie et al. 2005; Kuwabara et al. 2009). β-Catenin also accumulates in the cell bodies of postmitotic thalamic neurons on a constant basis and independently of signal transduction through Wnt receptors (Lucas et al. 1999; Wisniewska et al. 2010; Misztal et al. 2011; Pratt et al. 2012).

The LEF1/TCF family has four members in mammals: TCF7, TCF7L1, TCF7L2 (also known as TCF1, TCF3, and TCF4, respectively), and LEF1 (Arce et al. 2006; Archbold et al. 2012). They contain a β-catenin-binding domain at the *N*-terminus and a high-mobility group domain at the central part that recognizes the WWCAAAG DNA sequence, known as Wnt responsive element (WRE) (van de Wetering et al. 1997; van Beest et al. 2000; Barolo 2006; Hallikas et al. 2006; Chang et al. 2008). Despite the high sequence similarity among LEF1/TCF family members in these two domains, they display different promoter specificities within a particular cellular context. Moreover, the diversity of LEF1/TCF proteins is further increased by alternative promoter usage and extensive alternative splicing (Arce et al. 2006; Archbold et al. 2012). These isoforms can be categorized into several classes. One set of isoforms lacks the β-catenin-binding domain and acts as dominant-negative forms (Roose et al. 1999; Hovanes et al. 2000). Another group results from the alternative splicing of *C*-terminal exons, which encode an additional DNA-binding domain, the C clamp, found in some LEF1/TCFs (Van de Wetering et al. 1996; Duval et al. 2000; Hovanes et al. 2000; Nazwar et al. 2009; Weise et al. 2010; Vacik et al. 2011). This domain has been shown to make a contact with a type of GC-rich element, the so-called TCF-Helper sequence, located on either side of WRE. The C clamp dramatically increases the DNA-binding affinity and enables transcriptional regulation of a distinct set of the target genes (Hovanes et al. 2001; Hecht and Stemmler 2003; Atcha et al. 2007; Chang et al. 2008; Hoverter et al. 2012). Finally, the alternative splicing of other domains may modify the properties of LEF1/TCFs, e.g. affect their ability to act as either repressors or activators (Gradl et al. 2002; Liu et al. 2005; Ghogomu et al. 2006).

In contrast to the well-established roles of LEF1/TCF and β-catenin during development (Yamaguchi 2001; Logan and Nusse 2004; Ciani and Salinas 2005; Petersen and Reddien 2009; Freese et al. 2010; Niehrs 2010; Archbold et al. 2012), the potential functions of these proteins in the adult brain remain obscure. Several recent observations point to an important role of these factors in proper brain function and its pathologies. For example, variants of the *TCF7L2* gene have been associated with schizophrenia (Hansen et al. 2011; Alkelai et al. 2012), and anxiety-like phenotypes were observed in *Tcf7l2* haplo-insufficient mice (Savic et al. 2011), suggesting the involvement of *TCF7L2* in some psychiatric disorders. In addition, several other Wnt pathway components, such as *FZD3*, *GSK*-*3β*, *DKK4*, and *APC*, have been shown to be associated with susceptibility to schizophrenia (Cui et al. 2005; Kishimoto et al. 2008; Proitsi et al. 2008; Li et al. 2011), and many proteins mutated in sporadic autism are part of the β-catenin signaling network (O’Roak et al. 2012). A neural role of this pathway is further supported by the finding that LEF1/TCF can regulate genes that encode proteins implicated in neuronal excitability in the thalamus (Wisniewska et al. 2010), one of the regions that often exhibits abnormalities in schizophrenia (Byne et al. 2009; Cronenwett and Csernansky 2010; Pinault 2011; Guller et al. 2012). During embryonic development, *Tcf7l2* is primarily expressed in the thalamic and pretectal neuroepithelial domains at early stages of vertebrate development. This expression is subsequently extended to the midbrain and other forebrain regions during early steps of the specification of postmitotic neuroepithelial cells in all studied vertebrates (Cho and Dressler 1998; Ferran et al. 2007; Bluske et al. 2009; Quinlan et al. 2009; Merchán et al. 2011; Morona et al. 2011). However, expression data are much scarcer for postnatal stages. In the adult mouse brain, the *Tcf7l2* transcript has been described in the thalamus, pretectum, and some midbrain derivatives (Lee et al. 2009). During embryogenesis *Lef1* is detected in the thalamus pretectum and midbrain in mouse, chick and zebrafish (Bluske et al. 2009; Quinlan et al. 2009; Peukert et al. 2011), and is observed in the adult mouse and monkey thalamus (Jones and Rubenstein 2004; Shimogori et al. 2004). LEF1 protein was detected in postmitotic thalamic neurons in the zebrafish and adult rat (in the latter co-localizing with nuclear β-catenin) (Wisniewska et al. 2010; Misztal et al. 2011; Peukert et al. 2011). Therefore, as a first step to understand the physiological role of the LEF1/TCF transcriptional regulators in adult brain function, comprehensively examining their expression levels and alternative splicing variants and describing β-catenin and LEF1/TCF protein localization in detail are necessary. Here, we used quantitative polymerase chain reaction (qPCR) to measure the absolute expression levels of all LEF1/TCF genes in the cortex, thalamus, and midbrain from the late gestational stage to adulthood, distinguishing between full-length and dominant-negative isoforms. This was followed by an analysis of alternatively spliced forms of *Lef1* and *Tcf7l2* and their protein products in the embryonic and adult thalamus. Finally, we performed immunohistochemical staining to provide a detailed map of LEF1 and TCF7L2 protein distribution in cortical, thalamic, and mesencephalic regions in adult mice. We also established their co-localization with β-catenin and the neuronal marker NeuN. This study provided the first in-depth map of LEF1/TCF expression at postnatal stages.

## Materials and methods

### Animals

C57BL/6 mice were housed under standard conditions on a 12 h/12 h light/dark cycle (lights on at 7:00 a.m.) with food and water available ad libitum. All of the experimental procedures were approved by the Polish local Ethical Committee No. 1 in Warsaw.

### Antibody characterization

Anti-TCF4 (6H5-3) mouse monoclonal antibody was raised against amino acids 31-331 of human TCF7L2 (Millipore). Brain staining patterns of this antibody replicate the published *Tcf7l2* mRNA pattern determined by in situ hybridization (Lee et al. 2009). This antibody was previously shown to recognize different *C*-terminal mouse isoforms on immunoblots (bands 50–70 kDa) and by immunofluorescence (Weise et al. 2010).

Anti-TCF4 (N-20) goat polyclonal affinity-purified antibody was raised against a peptide that maps amino acids 1-50 in the *N*-terminus of TCF7L2 of human origin (Santa Cruz Biotechnology). The staining pattern is identical to mouse anti-TCF4 6H5-3 antibody. This goat antibody was previously shown to recognize different *C*-terminal mouse isoforms on immunoblots (bands 50–70 kDa) (Weise et al. 2010).

Anti-TCF4 (C48H11) rabbit monoclonal antibody was raised against a synthetic peptide that corresponds to residues that surround Leu330 of human TCF7L2 (Cell Signaling Technology). This antibody shows bands that correspond to known TCF7L2 isoforms on Western blots of mouse brain structures and colon nuclear fractions and HeLa cells that express recombinant TCF7L2 isoforms (Fig. 3e). This is consistent with a previous report (Vacik et al. 2011).

Anti-LEF1 (N-17) goat polyclonal antibody was raised against amino acids 1–50 mapped to the *N*-terminus of human LEF-1 (Santa Cruz Biotechnology). The antibody recognizes endogenous LEF1 protein on Western blots of whole-cell lysates from lymphoblastic lymphoma cell lines and immortalized T lymphocytes (manufacturer’s technical information). This antibody was previously shown to stain lung mesenchyme (Yin et al. 2008) and rat brain (Wisniewska et al. 2010). The observed staining pattern was consistent with previously reported in situ hybridization experiments in mouse brains (Jones and Rubenstein 2004; Shimogori et al. 2004).

Anti-LEF1 (C18A7) rabbit monoclonal antibody was produced by immunizing animals with a synthetic peptide that corresponds to residues that surround Pro82 of human LEF1 (Cell Signaling Technology). The antibody recognizes endogenous LEF1 protein, including isoforms that lack the *N*-terminus, on Western blots of total cell lysates from mouse thymocytes, DLD1 cells, and HCT15 cells (manufacturer’s technical information). This antibody was also used to detect endogenous and recombinant protein on Western blots (Hoeppner et al. 2011). In our Western blot analysis of nuclear fractions of mouse brain structures and the thymus and extracts of HeLa cells that express recombinant LEF-HA protein, we observed bands that were consistent with known isoforms (Fig. 2d).

Anti-β-catenin (H-102) rabbit polyclonal antibody was raised against amino acids 680–781 that mapped to the *C* terminus of β-catenin of human origin (Santa Cruz Biotechnology). This antibody recognizes the expected band around 92 kDa on Western blots from different brain structure homogenates and shows both membrane and nuclear staining of β-catenin on rat immunohistochemical brain sections (Wisniewska et al. 2010).

Anti-NeuN mouse monoclonal antibody was raised against purified cell nuclei from mouse brain. It reacts with most neuronal cell types and is frequently used as a pan-neuronal marker (Millipore). Immunohistochemical staining is primarily in the nuclei of the neurons. On Western blots, it recognizes two to three bands in the 46–48 kDa range.

### DNA constructs

The expression plasmids used for heterologous expression have been published previously. Mouse *Lef1* was a kind gift from Prof. Rudolf Grosschedl (Max Planck Institute of Immunobiology, Freiburg, Germany). *Tcf7l2*-E2, *Tcf7l2*-S2, *Tcf7*-B, and *Tcf7l1* were obtained from Prof. Andreas Hecht (Institute of Molecular Medicine and Cell Research, Freiburg, Germany). The *Tcf7l2*-S3 splice variant sequence (described in Weise et al. 2010) was amplified from the *Tcf7l2*-S2 plasmid by PCR using the 5′-ATTCTAGAATGCCGCAGCTGAACGG-3′ primer that incorporates the XbaI site and 5′-GCGGCCCCTGCAGTTTGTAGGTACCAA-3′ primer that incorporates the KpnI site. The PCR product was cloned into a pCG vector.

### HeLa cell transfection

HeLa cells (ATCC) were cultured according to the manufacturer’s instructions. A total of 1 × 10^6^ cells were seeded on a six-well plate 1 day before transfection, which was performed with 0.25 μg of expression plasmids together with 0.5 μg empty plasmid. Twenty microliters of OPTI-MEM medium (Invitrogen) and 2 μl of 1 mg/ml polyethylenimine (PEI) were added to the DNA, vortexed, and incubated for 15 min at room temperature. The mixture was then diluted with 120 μl of complete medium and added to the cells. The cells were further processed after 48 h.

### Protein extraction and immunoblotting

HeLa cells that overexpressed LEF1-HA and TCF7L2-E2 and TCF7L2-S3 isoforms were lysed in radioimmunoprecipitation assay buffer (50 mM Tris, pH 7.5, 150 mM NaCl, 1 mM ethylenediaminetetraacetic acid [EDTA], 1 % NP40, 0.5 % Na-deoxycholate, 0.1 % sodium dodecyl sulfate [SDS], and Complete protease inhibitor cocktail [Roche]), homogenized, briefly sonicated, and cleared by centrifugation at 12,000×*g* at 4 °C for 10 min.

For the preparation of nuclear extracts, the cortex, thalamus, and midbrain from embryonic (embryonic day 18.5 [E18.5]) and adult mice, adult gut, and adult thymus were dissected. The tissues were homogenized in ice-cold lysis buffer (50 mM Tris–HCl, pH 7.4, 5 mM MgCl_2_, 1 mM DTT, and Complete protease inhibitor cocktail [Roche]), followed by centrifugation at 800×*g* for 10 min. The supernatants were removed, and the pellets (i.e., nuclear fractions) were washed in lysis buffer and again centrifuged. The nuclear pellets were resuspended in hypotonic swelling buffer (20 mM HEPES, pH 7.9, 50 mM NaCl, 1.5 mM MgCl_2_, 0.2 mM EDTA, 1 mM DTT, and Complete protease inhibitor cocktail [Roche]) and incubated for 30 min with gentle rocking at 4 °C. The nuclear extracts were briefly sonicated, cleared by centrifugation at 9,000×*g* at 4 °C for 30 min, and stored at −80 °C until use.

The protein concentrations in cell lysates and nuclear extracts were determined using the Bradford Protein Assay kit (BioRad). For Western blotting, 20 μg of nuclear extracts and 1 μg of HeLa lysates were separated by electrophoresis on a 10 % SDS–polyacrylamide gel and transferred to a nitrocellulose membrane (Whatmann). The blots were blocked with 5 % dry non-fat milk powder in Tris-buffered saline with 0.5 % Tween 20 (TBST), washed 3 × 5 min with TBST, and incubated overnight at 4 °C with rabbit anti-LEF1 (1:1,000; C18A7, Cell Signaling Technology) and rabbit anti-TCF4 (1:1,000; C48H11, Cell Signaling Technology) antibodies diluted in 0.5 % milk in TBST. After three washes in TBST, membranes were probed with anti-rabbit secondary horseradish peroxidase (HRP)-conjugated antibody. Immunoreactivity was visualized with a chemiluminescent substrate solution that consisted of 0.1 M Tris–HCl, pH 8.6, 1.25 mM luminol, 0.23 mM p-Coumaric acid, and 0.0003 % H_2_O_2_.

### RNA isolation and cDNA preparation

Embryonic E16.5 to adult mouse brains were removed and placed on ice for the isolation of the cortex, thalamus and midbrain. The thalamus was dissected rostral to the superior colliculus in a line that pass approximately in the habenulo-interpeduncular tract, that is the caudal landmark of the thalamic region. Sectioned brain structures were immediately snap-frozen in liquid nitrogen and kept at −80 °C until RNA isolation. The RNA was isolated using a lipid-tissue designed kit (RNeasy for Lipid Tissue, Qiagen) and additionally treated with DNase (Qiagen). The RNA (2 μg) was transcribed to cDNA using the SuperScript III RNase H-Reverse Transcriptase kit (Invitrogen) according to the manufacturer’s instructions.

### Reverse-transcription quantitative polymerase chain reaction

The level of mRNA was examined using SYBR Green chemistry (Applied Biosystems) and probes designed using PrimerQuestSM on a 7900HT Fast Real-Time PCR System (Life Technologies). Reverse-transcription qPCR (RT-qPCR) was performed on cDNA synthesized from 20 ng of total RNA per point. The primers were designed to cover all known isoforms. The sequences of the used primers are listed in Table 1. Copy numbers were calculated using standard curves (10^2^–10^7^ copies) generated with serial dilutions of linearized plasmids that carry *Lef1*, *Tcf7*, *Tcf7l1*, and *Tcf7l2* cDNAs.

**Table 1:** Primer sequences

	Sequence
Primers for RT-PCR	
Lef1 Ex5f	5′-ACCCTCCAGCTCCTGAAATC-3′
Lef1 Ex7r	5′-TTAGGTCACTGTCCGTGTGG-3′
Lef1 Ex10f	5′-CGCGAGACAATTATGGCAAG-3′
Lef1 Ex12r	5′-TTCAACAAGCTTCCATCTCCA-3′
Tcf7l2 Ex3f	5′-GCCAAGAGGCAAGATGGAG-3′
Tcf7l2 Ex5r	5′-ACGAGCATCCTTGAGGGTTT-3′
Tcf7l2 Ex6r	5′-ACGTGATGAGAGGCGTGAGT-3′
Tcf7l2 Ex11f	5′-CCAGGGAAGAACAGGCAAAAT-3′
Tcf7l2 E17r	5′-GGGGAGGCGAGTCTAGTAAGC-3′
Tcf7l2 E12r	5′-AAGAGAAAAAGAGACAAGCAGCC-3′
Primers for RT-qPCR	
Lef1f	5′-TGGCATCCCTCATCCAGCTATTGT-3′
Lef1r	5′-TGAGGCTTCACGTGCATTAGGTCA-3′
Lef1 FLf	5′-AACTCTGCGCCACCGATGAGATGAT-3′
Lef1 FLr	5′-ATGACTTGATGTCGGCTAAGTCGC-3′
Tcf7f	5′-AGAGAAGGAGGCTAAGAAGCCAGT-3′
Tcf7r	5′-ACTCAGCAATGACCTTGGCTCTCA-3′
Tcf7l1f	5′-GGAGCCGGGGCAACCAGTG-3′
Tcf7l1r	5′-CATCCTGGGGCCTTCTCACTTC-3′
Tcf7l2f	5′-GGTGGCCGAATGCACATTGAAAGA-3′
Tcf7l2r	5′-TTTGCCTGTTCTTCCCTGGACA-3′
Tcf7l2 FLf	5′-AACTCCTCGGCGGAAAGGGATTTA-3′
Tcf7l2 FLr	5′-TTGGCCGCTTCTTCCAAACTTTCC-3′

### Splicing analysis and reverse-transcription polymerase chain reaction

To investigate all possible isoforms generated from mouse *Lef1* and *Tcf7l2*, we combined information from previous publications (Carlsson et al. 1993; Roose et al. 1999; Duval et al. 2000; Hovanes et al. 2000; Pukrop et al. 2001; Young et al. 2002; Nazwar et al. 2009; Weise et al. 2010; Vacik et al. 2011), available estimated sequence tags and mRNAs from National Center for Biotechnology databases, and Ensembl genome annotations for mouse, human, and zebrafish. Each potential alternative splicing event was manually annotated in the mouse genomic sequence (Figs. 2a, 3a), and different sets of primers were designed to cover all possible alternative splicing events. In general, primers were designed on adjacent constitutive exons that spanned the entire alternative region, with the exception of investigating the possible inclusion/exclusion of exons 5b and 12 in Tcf7l2. For primer design, we used Primer3 software, requiring that the ratio between short and long isoforms did not exceed 1:2, when possible, to minimize biased PCR amplification of short amplicons (Rukov et al. 2007). For RT-PCR, cDNA according to 50 ng RNA was amplified using the primers listed in Table 1 and *Taq* DNA polymerase (EURx Ltd). PCR products were separated by electrophoresis on 2.5 % agarose gels.

**Table 2:** Relative regional expression of LEF1, TCF7L2, and nuclear β-catenin in the mouse forebrain and midbrain

Region	LEF1	TCF7L2	Nuclear β-catenin	Region	LEF1	TCF7L2	Nuclear β-catenin
Cerebral cortex	−	−*	−	Thalamus (continued)			
Corpus callosum	−	−*	−	Reuniens	++	+++	+++
Hippocampal formation	−	−*	−	Mediodorsal	+++	++	+++
Entorhinal cortex, lateral	++/+++	−	−	Ventral anterolateral	+++	++++	+++
Striatum	−	−*	−	Ventral Medial	++++	+++	+++
Pallidum				Ventral posterolateral	+++	++	+++
Ventral pallidum	−	+	+	Ventral posteromedial	+++	+++	+++
Substantia innominata	−	+	+	Laterodorsal	+++	+++	+++
Magnocellular preoptic nucleus	−	+	+	Posterior	+++	++++	+++
Nucleus of the horizontal limb of the diagonal band	−	+	+	Lateral posterior	+++	++++	+++
Preoptic area	−	+	+	Parafascicular	±	+++	+++
Prethalamus				Submedius	+++	+++	+++
Reticular	−	−	−	Dorsal lateral geniculate	+++	+++	+++
Pregeniculate	−	+	+	Medial geniculate	+++	+++	+++
Zona incerta	−	−	−	Pretectal region	−	++	++
Epithalamus				Periaqueductal gray			
Medial habenula	∓	++	++++	Dorsomedial	+	+	++
Lateral habenula	−	−	−	Dorsolateral	+	++	++
Thalamus				Lateral	−	++	+
Paraventricular	−	+++	+++/++++	Ventrolateral	−	−	−
Parataenial	+	+++	+++	Superior colliculus			
Anteroventral	++	+++	+++	Zona layer	++	−	++
Anteromedial	++	+++	++	Superficial gray layer	++	−	++
Anterodorsal	+	+++	++/+++	Optic nerve layer	++	+	++
Central medial	+	+++	++	Intermediate layers	++	+	++
Rhomboid	++	+++	+++	Deep layers	++	+	++
Paracentral	+	+++	+++	Interpeduncular medial nucleus	−	+	+
Intermediodorsal	+	+++	+++	Inferior colliculus	−	++/+++	−

The relative intensity of nuclear immunostaining or number of cells that express LEF1, TCF7L2, and β-catenin in the specified brain regions is summarized. The intensity of nuclear labeling was estimated as highest (++++), high (+++), moderate (++), low (+), background/no labeling (−), or small number of none-neuronal cells stained (−*)

### RNA-seq analysis

For RNA-seq analysis, we prepared Bowtie sequence libraries with all possible exon–exon junctions for both genes and mapped RNA-seq from mouse brain, liver, kidney, and heart (Brawand et al. 2011) using bowtie with –m 1 –v 2 parameters. The inclusion level for each alternative sequence and sample was determined as previously described (Ellis et al. 2012).

### Perfusion of mice and brain sectioning

Male mice (P60) were deeply anesthetized with an intraperitoneal injection of Morbital and transcardially perfused with a 0.1 M phosphate-buffered saline (PBS) solution, pH 7.4, followed by 4 % paraformaldehyde in 0.1 M PBS, pH 7.4. Dissected brains were postfixed overnight and further cryoprotected overnight with 30 % sucrose. The brains were coronally sectioned at 40 μm with a cryostat.

### Immunocytochemistry

Free-floating sections were first quenched with 0.3 % H_2_O_2_ for 10 min and rinsed in PBS that contained 0.2 % Triton X-100 (PBST; 3 × 10 min), blocked for 30 min with 3 % serum of the appropriate species in PBST, and incubated with primary antibodies (mouse anti-TCF4, 1:1,000, Millipore; goat anti-LEF-1, 1:200, Santa Cruz Biotechnology; rabbit anti-β-catenin, 1:1,000, Santa Cruz Biotechnology) diluted in 1 % serum in PBST. After washing, the sections were incubated with biotinylated secondary anti-rabbit, anti-goat, or anti-mouse antibodies (1:500; Vector Labs). Staining was visualized by a reaction with 3,3′-diaminobenzidine substrate using an ABC kit (Vector Labs). When using anti-mouse primary antibody, the Vector M.O.M. Immunodetection Kit (Vector Labs) was used. Photographs were taken with a Nikon Eclipse 80i microscope.

### Immunofluorescence

Free-floating sections were blocked for 30 min with 3 % serum of the appropriate species in PBST and incubated with primary antibodies (goat anti-TCF4, 1:500, Santa Cruz Biotechnology; goat anti-LEF-1, 1:100, Santa Cruz Biotechnology; rabbit anti-β-catenin, 1:500, Santa Cruz Biotechnology; mouse anti-NeuN, 1:500, Millipore) diluted in 1 % serum in PBST. For multiple fluorescent immunostainings, sections were incubated with combinations of appropriate secondary antibodies conjugated with Alexa Fluor 488 and 568 dyes (1:500; Molecular Probes) for 1 h before they were mounted onto slides with Vectashield medium (Vector Labs). Photographs were taken with a Nikon Eclipse 80i microscope.

### Image processing

Digital microscopic images were slightly modified to optimize image resolution, brightness, and contrast using Adobe Photoshop CS4 software to best represent the immunohistochemistry and immunofluorescence observed with the microscope.

## Results

### Expression levels of Lef1, Tcf7, Tcf7l1, and Tcf7l2 mRNA in the developing and adult cortex, thalamus, and midbrain

We analyzed the expression of LEF1/TCF transcription factors in the regions where their expression has been previously reported during late development, i.e., in the thalamus and midbrain. In addition, we included the cortex because of the thalamocortical and corticothalamic relationships described in the developing (Pratt et al. 2012) and adult (Steriade 1997) brain.

**Fig. 1:**
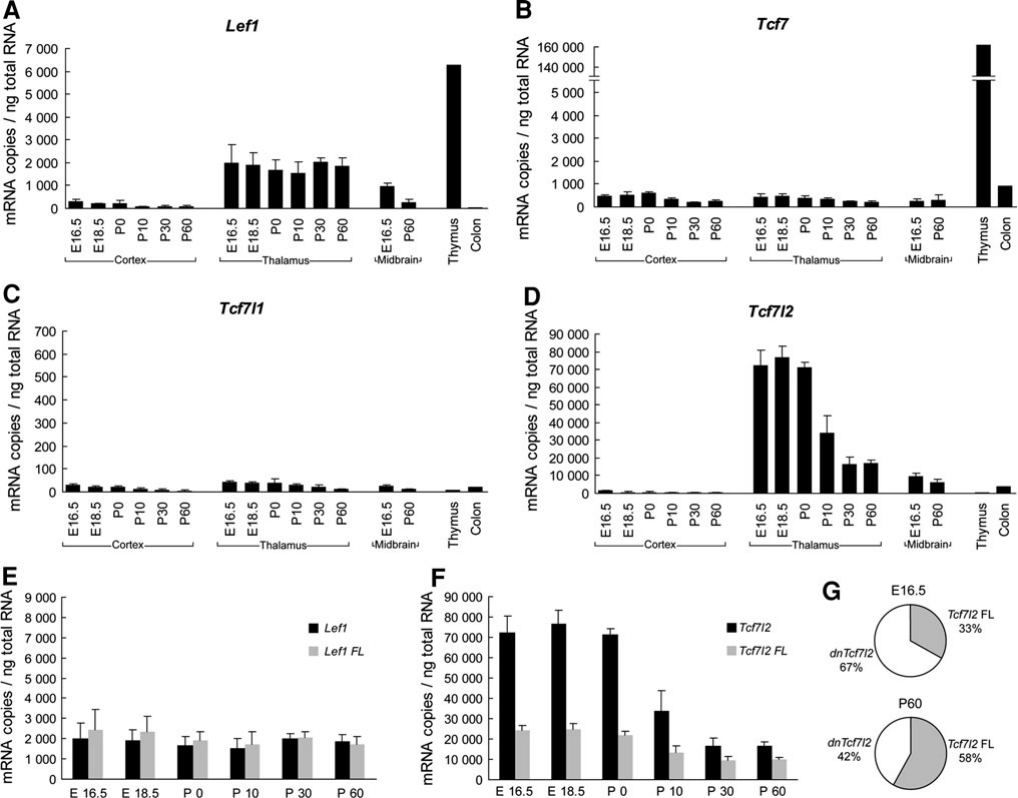
Expression analysis of the *Lef1*, *Tcf7, Tcf7l1,* and *Tcf7l2* genes. mRNA copy numbers of **a** *Lef1*, **b** *Tcf7*, **c** *Tcf7l1*, and **d** *Tcf7l2* were measured in the developing and adult cortex, thalamus, and midbrain and adult thymus and colon. The results are shown as mRNA copy number per 1 ng of total RNA (estimated to correspond to ~40 brain cells). Copy numbers were calculated using standard curves generated with serial dilutions of linearized plasmids that carry *Lef1*, *Tcf7*, *Tcf7l1*, and *Tcf7l2* cDNAs. The data are expressed as mean ± SD (*n* = 3), except for the thymus and colon. Comparisons between copy numbers of full-length (FL) and truncated isoforms of **e** *Lef1* and **f** *Tcf7l2* in the developing thalamus were made using different primer pairs, the positions of which are marked in Figs. 2a and 3a, respectively. The data are expressed as mean ± SD (*n* = 3). **g** The pie chart indicates the percentage contribution of full-length (*FL*) and dominant-negative (*dn*) *Tcf7l2* isoforms in the embryonic and adult thalamus

To provide accurate measurements of mRNA levels, we first performed RT-qPCR and used standard curves to calculate mRNA copy numbers. The level of mRNA of every LEF1/TCF gene (*Lef1*, *Tcf7*, *Tcf7l1*, and *Tcf7l2*) was quantified in the embryonic (E16.5 and E18.5), postnatal (postnatal day 0 [P0], P10, and P30), and young adult (P60) thalamus and cortex, as well as in the E16.5 and P60 midbrain (Fig. 1a–d). For comparison, we isolated RNA from the thymus, where high levels of *Lef1* and *Tcf7* are present in T lineage lymphocytes (Waterman et al. 1991; Van de Wetering et al. 1996), and colon, where *Tcf7* and *Tcf7l2* are expressed in epithelial stem cells throughout life (Korinek et al. 1998a; Roose et al. 1999). As a reference frame, we considered less than 1,000 mRNA molecules per 1 ng of total RNA as a low expression level, between 1,000 and 10,000 as a moderate expression level, and >10,000 as a high expression level. For further estimations, we assumed that 1 ng of total RNA comes from ~40 brain cells, including both neurons and astrocytes (Norton and Poduslo 1971).

The mRNA level of *Lef1* was moderate in the developing and adult thalamus (estimated to be ~50 copies/cell) and developing midbrain and low in the adult midbrain and developing and adult cortex (Fig. 1a). Interestingly, whereas *Lef1* mRNA levels appeared to significantly decrease in the midbrain during development, they remained constant in the thalamus throughout all of the studied stages (Fig. 1a). In the case of *Tcf7*, mRNA expression was low in all of the samples (5–10 copies/cell), with the exception of the thymus, where it was extremely high (Fig. 1b). Virtually no expression of *Tcf7l1* (<1 copy/cell) was found in any of the analyzed tissue (Fig. 1c). In contrast, *Tcf7l2* mRNA level was high in the embryonic and perinatal thalamus (~2,000 copies/cell). Although it gradually decreased approximately fourfold during development, it still remained high (~400 copies/cell) in the adult (Fig. 1d). In the midbrain and E16.5 cortex, *Tcf7l2* mRNA levels were moderate and then gradually decreased to a low level in the latter.

**Fig. 2:**
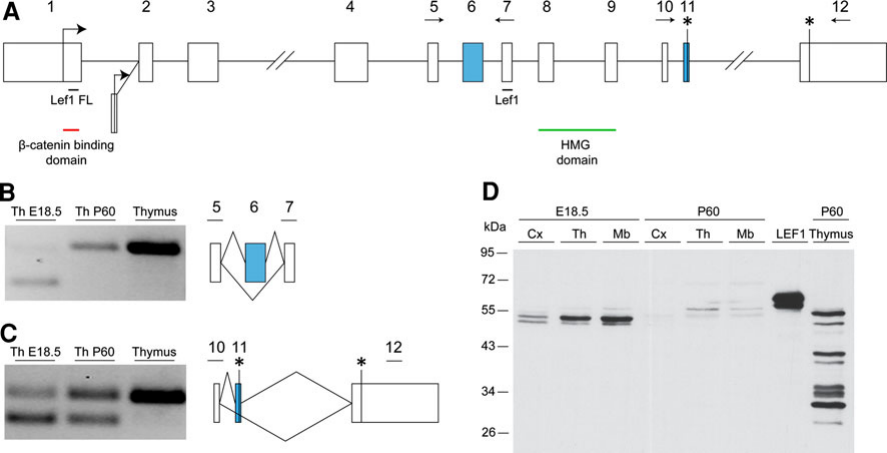
Analysis of alternative splicing of *Lef1* in the mouse thalamus. **a** Representative intron–exon structure of the *Lef1* gene. Long introns are represented by a *double slash*. *Blue boxes* indicate the alternatively spliced exons. Important protein domains are marked by *red* and *green lines* under the exons by which there are encoded. The position and orientation of the primers used for RT-PCR are indicated by horizontal arrows. The *horizontal lines* with “Lef1 FL” and “Lef1” indicate the regions of the gene that were tested in the RT-qPCR analysis in Fig. 1. **b** RT-PCR expression analysis of alternative splicing of exon 6. **c** RT-PCR expression analysis of alternative splicing of exon 11. **d** Western blot analysis of the endogenous LEF1 protein in the E18.5 and adult cortex (*Cx*), thalamus (*Th*), and midbrain (*Mb*) and adult thymus. Fast-migrating polypeptides in the thymus may represent alternative forms of LEF1, degradation products, or other nonspecific proteins. As a control, HeLa cells transfected with LEF1-HA expression plasmids were used

**Fig. 3:**
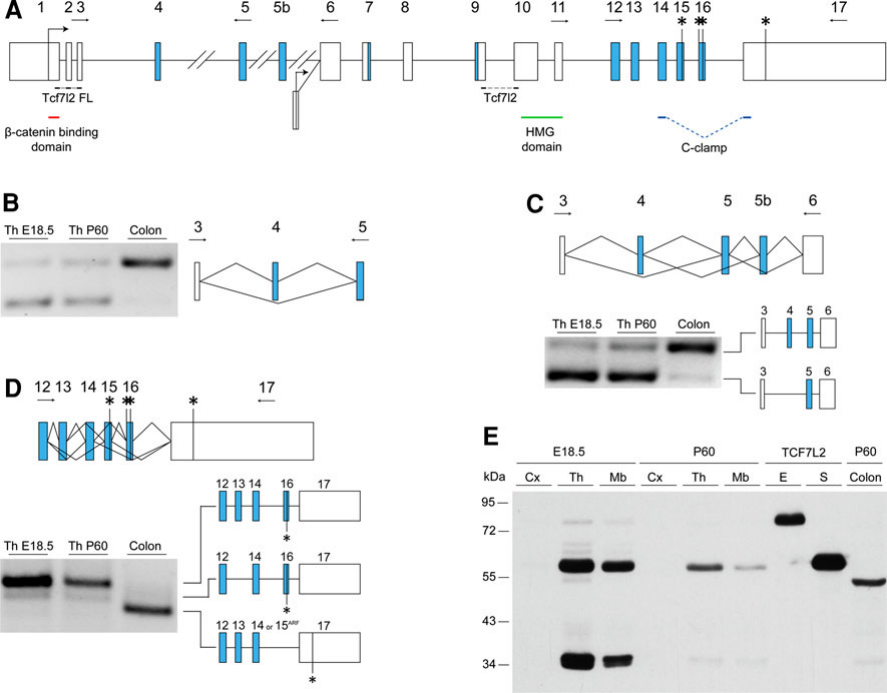
Analysis of alternative splicing of *Tcf7l2* genes in the mouse thalamus. **a** Representative intron–exon structure of the *Tcf7l2* gene. Long introns are represented by a *double slash*. Blue boxes indicate the alternatively spliced exons. Important protein domains are marked by *red*, *green* and *blue lines* under the exons by which there are encoded. The position and orientation of the primers used for RT-PCR are indicated by *horizontal arrows*. The *horizontal lines* with “Tcf7l2 FL” and “Tcf7l2” indicate the regions of the gene that were tested in the RT-qPCR analysis in Fig. 1. **b** RT-PCR expression analysis of alternative splicing of exon 4. **c** RT-PCR expression analysis of alternative splicing of exon 5. **d** RT-PCR expression analysis of alternative splicing of exons 13–16. Colon transcript resulted from inclusion of exon 14 or 15 translated from different alternative reading frame (*ARF*). **e** Western blot analysis of the endogenous TCF7L2 protein in the E18.5 and adult cortex (*Cx*), thalamus (*Th*), and midbrain (*Mb*) and adult colon. As a control, HeLa cells transfected with TCF7L2-E2 and TCF7L2-S3 expression plasmids were used

*Lef1* and *Tcf7l2* genes have been previously shown to contain alternative promoters located in introns 2 and 5, respectively, which generate truncated mRNA isoforms that encode dominant-negative proteins (Hovanes et al. 2000; Vacik et al. 2011). We estimated the specific contribution of these truncated forms to the total pool of mRNAs from each gene during thalamus development. For each gene, a pair of primers that specifically target the most-upstream canonical first exons was designed (Figs. 2a, 3a). In the case of *Lef1*, all of the transcripts in the thalamus corresponded to full-length isoforms because the estimated levels were similar to the full-length-specific primers and primers common to all isoforms (Fig. 1e). Accordingly, RNA-seq data from the adult mouse brain supported the use of only the upstream promoter in this tissue. The truncated *Tcf7l2* mRNA was the predominant isoform in the embryonic thalamus (Fig. 1f) and constituted almost 70 % of all of the estimated mRNA molecules for this gene (Fig. 1g; this estimation was made by subtracting the short form-specific RT-qPCR measurement from the value obtained with a pair of primers common to all isoforms). The contribution of this dominant-negative isoform decreased by approximately 25 % during postnatal development but was still significantly high (Fig. 1g). The RNA-seq analysis of the whole adult brain supported the expression of the truncated *Tcf7l2* isoform from the alternative promoter located in intron 5.

In summary, *Lef1* and *Tcf7l2* mRNAs were the predominant members of the LEF1/TCF family in the thalamus and midbrain, whereas all of the members exhibited low expression in the cortex. Moreover, the truncated, dominant-negative *Tcf7l2* isoforms were the most abundant isoforms in the embryonic thalamus but not in the adult thalamus, while *Lef1* was expressed only as full-length isoforms at all of the studied stages.

### Identification of Lef1 splice variants in the embryonic and adult mouse thalamus

The LEF/TCF family exhibits not only alternative promoter usage but also extensive alternative splicing, giving rise to isoforms with unique specificities for promoter recognition and activation (see “Introduction”). Therefore, we also analyzed the splicing patterns of *Lef1* and *Tcf7l2* in the developing and adult brain. Given that the expression of these genes was the highest in the thalamus, we chose this region for analysis and compared it with the adult thymus and colon where *Lef1* and *Tcf7l2* are highly expressed, respectively. For this purpose, we reverse transcribed RNA isolated from the above tissues and PCR-amplified specific sequences of the *Lef1* and *Tcf7l2* cDNA with primers designed to span specifically the alternative splicing regions. We also analyzed RNA-seq data from different adult tissues, including whole brain, by measuring reads that span alternative exon–exon junctions and 3′ and 5′ alternative splice sites.

An in-depth analysis of the mouse *Lef1* intron–exon structure revealed two alternative first exons and two alternative splicing events (Fig. 2a) conserved to the human gene (Hovanes et al. 2000). The first alternative splicing event resulted in the skipping of exon 6 (Fig. 2b), which overlaps with an important regulatory domain (Carlsson et al. 1993; Mallory et al. 2011). This alternative splicing event showed a dramatic regulatory switch during development. Exon 6 was mostly skipped in the E18.5 thalamus, and nearly all of the transcripts include the exon by P60. Similarly, the adult thymus exhibited nearly no skipping of exon 6. We then investigated the second alternative exon (exon 11) located at the 3′ region of the *Lef1* gene (Fig. 2c). The inclusion of exon 11 causes a frame shift in the transcript, generating a slightly shorter isoform because of the introduction of a premature stop codon [short and long forms correspond to N and B isoforms in humans, respectively (Hovanes et al. 2000)]. Although less pronounced than for exon 6, exon 11 also exhibited strong developmental regulation, with increasing inclusion of this exon from P18.5 to P60 in the thalamus. The adult thymus exhibited no exon skipping. Consistent with the RT-PCR results, RNA-seq data from the adult brain showed high inclusion levels for both exons 6 and 11 (96 and 69 %, respectively).

To determine whether these alternative splicing events have an impact at the protein level, we performed a Western blot analysis with nuclear extracts from the embryonic and adult cortex, thalamus, and midbrain and adult thymus (Fig. 2d). In the embryonic tissues, we observed two ~53 to 54 kDa protein bands that corresponded to isoforms generated by the alternative splicing of exon 11. The more slowly migrating band, which was more prominent in the thalamus and midbrain than in the cortex, represented the long protein isoform that resulted from exon 11 exclusion. The band with higher electrophoretic mobility corresponded to the short protein isoform that included exon 11. Consistent with the RT-PCR analysis, the inclusion of exon 6 was widespread in the adult thalamus and midbrain, resulting in an increase in the molecular weight of the LEF1 protein. In addition, we observed a downregulation of LEF1 protein levels in the P60 thalamus compared with the E18.5 thalamus, in contrast to generally constant mRNA levels. Finally, we did not detect dominant-negative LEF1 isoforms (~37 kDa) in any of the analyzed brain regions, in contrast to the adult thymus. In summary, we observed a developmentally coordinated switch in the regulation of *Lef1* isoform composition, with increased inclusion of both exons 6 and 11, and also downregulation at the protein level.

### Identification of Tcf7l2 splice variants in the embryonic and adult mouse thalamus

*Tcf7l2* can give rise to diverse proteins by combining several alternatively spliced exons that are scattered throughout the entire length of the gene (Fig. 3a). We first determined the inclusion of exon 4, which encodes part of a domain involved in protein–protein interactions with repressors and activators (Arce et al. 2006; Archbold et al. 2012) and whose exclusion was shown to have dampening effects on *Tcf7l2* transactivation capacity (Weise et al. 2010). This exon had clear tissue-specific regulation, with both E18.5 and adult thalamus samples showing only ~25 % inclusion, whereas nearly all transcripts expressed in the colon included exon 4 (Fig. 3a). This difference is consistent with the RNA-seq data, in which inclusion in the liver and kidney was 40 and 20 % higher, respectively, than in the whole brain. We next investigated the alternative splicing of exon 5, which has been reported to be alternatively spliced in zebrafish (Young et al. 2002), and a previously uncharacterized exon between exons 5 and 6 (exon 5b in Fig. 3a). We used primers that spanned the constitutive exons 3 and 6, yielding several RT-PCR products (Fig. 3c). We observed the inclusion of exon 5 in both examined tissues but found no support for the inclusion of exon 5b. We then used RNA-seq to analyze the use of two alternative splice site choices at exon 7 (alternative 5′ splice site) and exon 9 (alternative 3′ splice site), whose alternative products were too short to be distinguished by RT-PCR. In the case of exon 7, the 12-nucleotide alternative sequence was included in 80 % of the transcripts in whole adult brain and nearly all of the transcripts in the liver and kidney (~95 %). The 15-nucleotide sequence of exon 9 was excluded in 98 % of the transcripts in the brain, with slightly higher inclusion levels in the other tissues (25–27 %). Finally, we investigated transcripts generated at the 3′ site of *Tcf7l2*. We first tested whether exon 12 was alternatively spliced, as predicted in the Ensembl annotations. Primer pairs that spanned exons 11 and 17 showed no evidence of the exclusion of exon 12 in the studied tissues and developmental stages (data not shown). We then focused on the combinatorial diversity generated by alternative splicing of exons 13-16, which generates isoforms with different C termini, namely TCF7L2-E harboring complete C clamp, TCF7L2-S with incomplete version of it and TCF7L2-M without C clamp (Weise et al. 2010). We examined the RT-PCR products obtained from primers that target exons 12 and 17 (Fig. 3d). The pattern of the RT-PCR products from the thalamus showed that the transcript that included exons 13, 14, and 16 was the most prevalent isoform in the thalamus. This observation was confirmed by sequencing the RT-PCR products. This combination of exons, when translated, gives rise to a TCF7L2-S isoform, i.e. with a truncated C clamp. In contrast, the most abundant transcripts in the colon resulted from the exclusion of exons 13 and 16 and inclusion of either exon 14 or 15, which give rise to TCF7L2-E isoforms that have a complete C clamp. These results are consistent with the RNA-seq data, which showed no inclusion of exons 13 and 16 for the liver or kidney and ~40 % inclusion of exon 13 in the brain. Therefore, the inclusion of exons 13 and 16 appeared to be highly tissue specific. To detect the endogenous proteins in the brain tissues, we performed a Western blot analysis using an antibody that recognizes all of the isoforms of TCF7L2 (Fig. 3e). In the embryonic thalamus and midbrain, we observed two predominant bands. The more slowly migrating 58 kDa corresponded to the TCF7L2-S isoforms, whereas the more rapidly migrating 35 kDa band matched the molecular weight of the dominant-negative TCF7L2-S isoform. Consistent with the RT-PCR data, we did not find any developmental change in splicing composition. However, in the adult thalamus and midbrain, the level of the full-length TCF7L2-S isoform proteins was reduced but still easy to detect, while the dominant-negative TCF7L2-S isoform was barely detected. In the cortex, we did not observe any of the TCF7L2 isoforms at any of the analyzed stages.

In summary, in contrast to the coordinated temporal regulation of *Lef1* alternative splicing, *Tcf7l2* events showed remarkable tissue-specific coordinated regulation, with the predominant isoforms in the thalamus lacking exon 4 and including exons 13, 14, and 16 translated to TCF7L2 with a truncated C clamp. Despite the lack of temporal regulation of *Tcf7l2* alternative splicing, we observed a change in alternative promoter usage, with full-length isoforms, i.e., the isoforms that contain a sequence for the β-catenin-binding domain, predominating in the adult thalamus.

### Distribution of LEF1, TCF7L2, and nuclear β-catenin in the adult mouse brain

The final aim of our study was to investigate the fine-scale spatial protein distribution of LEF1/TCF transcription factors in the adult mouse brain (P60). LEF1 and TCF7L2 proteins were observed in the thalamus and midbrain by Western blot. To determine their spatial and cell-specific distribution with much finer detail, we performed immunohistochemical staining of mouse brain sections with anti-LEF1 and anti-TCF7L2 antibodies (summarized in Table 2). In parallel, we stained the sections with a β-catenin-specific antibody to examine the subcellular localization of β-catenin and its co-localization with LEF1 and TCF7L2.

**Fig. 4:**
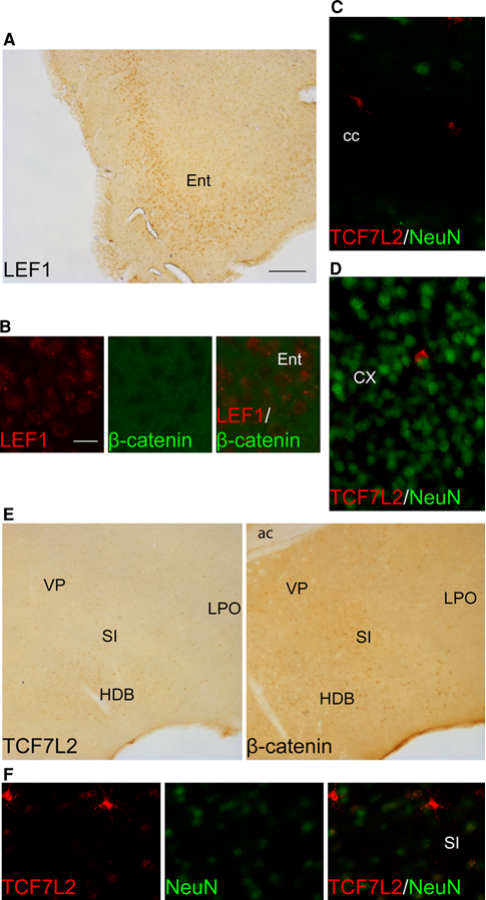
Distribution of LEF1, TCF7L2, and β-catenin proteins in the telencephalon. **a**, **b** LEF1 is highly expressed in the entorhinal cortex and does not co-localize with nuclear β-catenin. **c** Single TCF7L2 non-neuronal cells are found in the corpus callosum and cerebral cortex. **e** The expression of TCF7L2 and nuclear β-catenin is found in the substantia innominata and neighboring structures in **f** neuronal and non-neuronal cells. *ac* anterior commissure; *cc* corpus callosum; *CX* cerebral cortex; *Ent* entorhinal cortex; *HDB* nucleus of the horizontal limb of the diagonal band; *LPO* lateral preoptic area; *SI* substantia innominata; *VP* ventral pallidum. *Scale bar* 200 μm for **a** and **e**. *Scale bar* 25 μm in **b**, **c**, **d**, and **f**

In the telencephalon, widespread LEF1 expression was observed only in the lateral division of the entorhinal cortex, mostly at the level of layer V (Fig. 4a). Double immunofluorescent staining of LEF1 and β-catenin did not show nuclear β-catenin in these cells (Fig. 4b).

In the corpus callosum (cc), other white matter structures, and throughout the cerebral cortex, hippocampal formation, and striatum, we observed several scattered cells with high levels of TCF7L2 protein but a non-neuronal phenotype, determined by double immunofluorescence with the neuronal marker NeuN (Fig. 4c, d), which could be the previously described oligodendrocyte lineage cells (Fancy et al. 2009; Ye et al. 2009) In the ventral pallidum (VP), horizontal limb of the diagonal band, substantia innominata, and preoptic area, few cells were found that co-localized TCF7L2 and nuclear β-catenin (Fig. 4e). Double immunofluorescent staining with NeuN showed that this population of TCF7L2-positive cells contained both neuronal and non-neuronal cells (Fig. 4f).

**Fig. 5:**
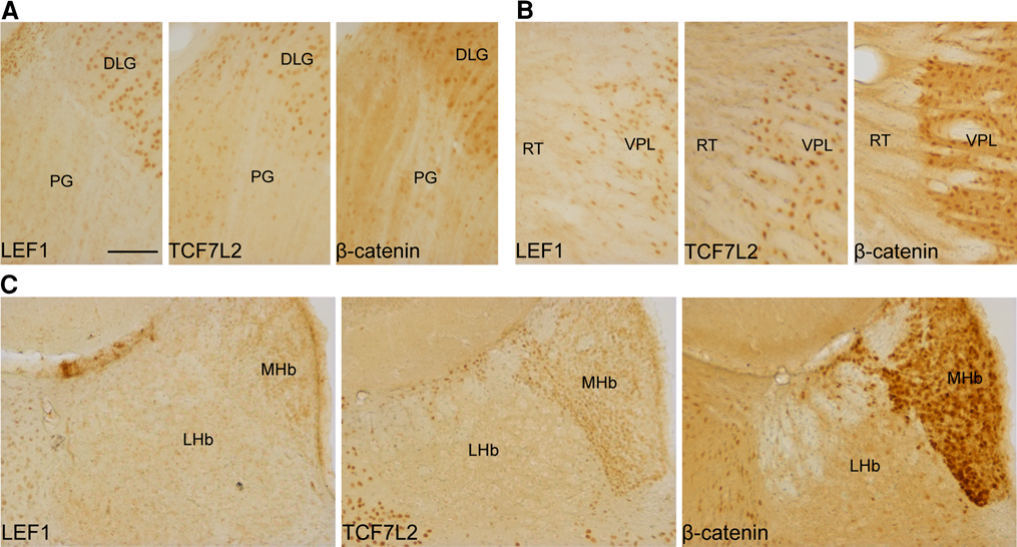
Distribution of LEF1, TCF7L2, and β-catenin proteins in the adult prethalamus and epithalamus. **a** TCF7L2 and nuclear β-catenin are present in the subsets of cells in the pregeniculate nucleus. **b** A clear border is seen between LEF1-, TCF7L2-, and nuclear β-catenin-positive cells between the thalamus and reticular thalamic nucleus. **c** In the habenula, LEF1, TCF7L2, and nuclear β-catenin are present only in the medial part. *DLG* dorsal lateral geniculate; *LHb* lateral habenula; *MHb* medial habenula; *PG* pregeniculate nucleus; *RT* reticular thalamic nucleus; *VPL* ventral posterolateral thalamic nucleus. *Scale bar* 100 μm

In the prethalamus, we observed nuclear labeling of TCF7L2 and β-catenin in some cells of the pregeniculate nucleus (formerly ventral lateral geniculate), with a higher density of stained cells in the magnocellular part (Fig. 5a). No staining of LEF1, TCF7L2, or nuclear β-catenin was observed in the reticular thalamic nucleus (Fig. 5b). In the epithalamus, LEF1 was detected at relatively low levels, mostly in the medial habenula (Fig. 5c). Nuclear TCF7L2 and β-catenin were observed in a high number of cells in the medial habenula but were not detected in the lateral habenula.

**Fig. 6:**
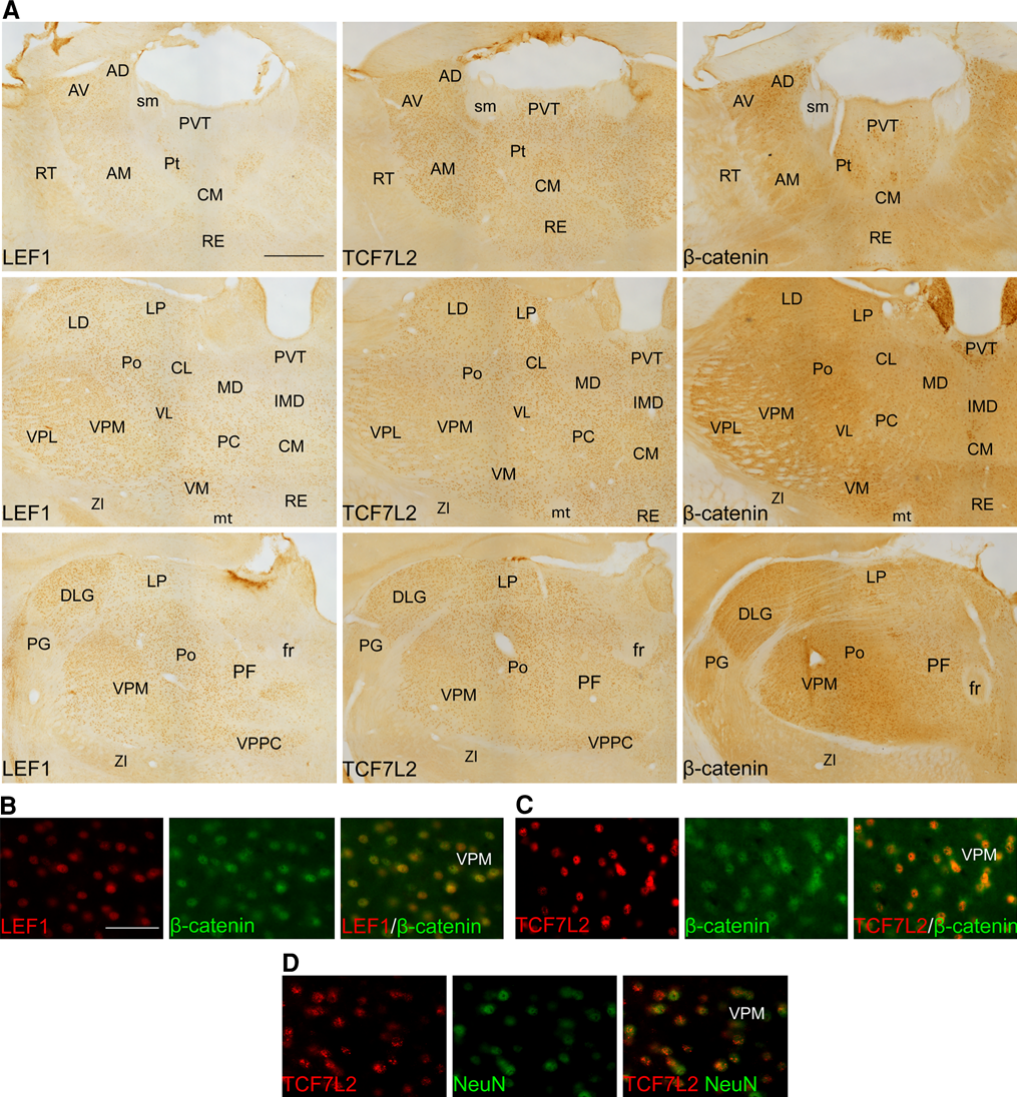
Distribution of LEF1, TCF7L2, and β-catenin proteins in the adult thalamus. **a** Photomicrographs show the localization of LEF1, TCF7L2, and nuclear β-catenin at different rostro-caudal levels of the thalamus. Double immunofluorescent staining shows that in thalamic cells that express **b** LEF1 and **c** TCF7L2 exhibit nuclear localization of β-catenin. **d** The colabeling with NeuN confirmed the neuronal phenotype of TCF7L2-poitive cells. For abbreviations, see list. *Scale bar* 200 μm for **a**. *Scale bar* 50 μm for **b**–**d**

Consistent with the RT-qPCR analyses, the thalamus was the region with the highest expression of both transcription factors and nuclear β-catenin. LEF1 was detected in virtually all thalamic nuclei, although with different relative intensities or numbers of cells that express the protein (Fig. 6a). LEF1 expression was easily detected in all ventral, mediodorsal, posterior, lateral posterior, laterodorsal, medial nuclei and dorsal lateral geniculate nucleus. The expression was relatively less visible in anteroventral, anteromedial, reunions, and rhomboid nuclei. By contrast, the protein was not detected in rostral paraventricular thalamic nucleus and in lateral part of parafascicular nucleus. Similarly, the anterodorsal nucleus and rostral intralaminar nuclei (central medial, paracentral, central lateral, and intermediodorsal) were stained at a relatively low level. In the case of TCF7l2, the intensity of the staining was consistently high across all thalamic derivatives (Fig. 6a), with only a relatively lower signal in mediodorsal and ventral posterolateral nuclei. Lateral posterior and posterior nuclei exhibited the highest relative intensity among all of the brain regions analyzed. Finally, nuclear β-catenin staining was seen in nearly all thalamic nuclei (Fig. 6a). The intensity of the labeling was particularly high in the caudal part of the paraventricular nucleus, similar to the observations in the medial habenula. Staining in the anteromedial and centromedial nuclei was relatively moderate. Double immunofluorescent staining showed that LEF1- and TCF7L2-positive cells were also β-catenin positive (Fig. 6b, c), and co-labeling with NeuN confirmed their neuronal phenotype (Fig. 6d).

**Fig. 7:**
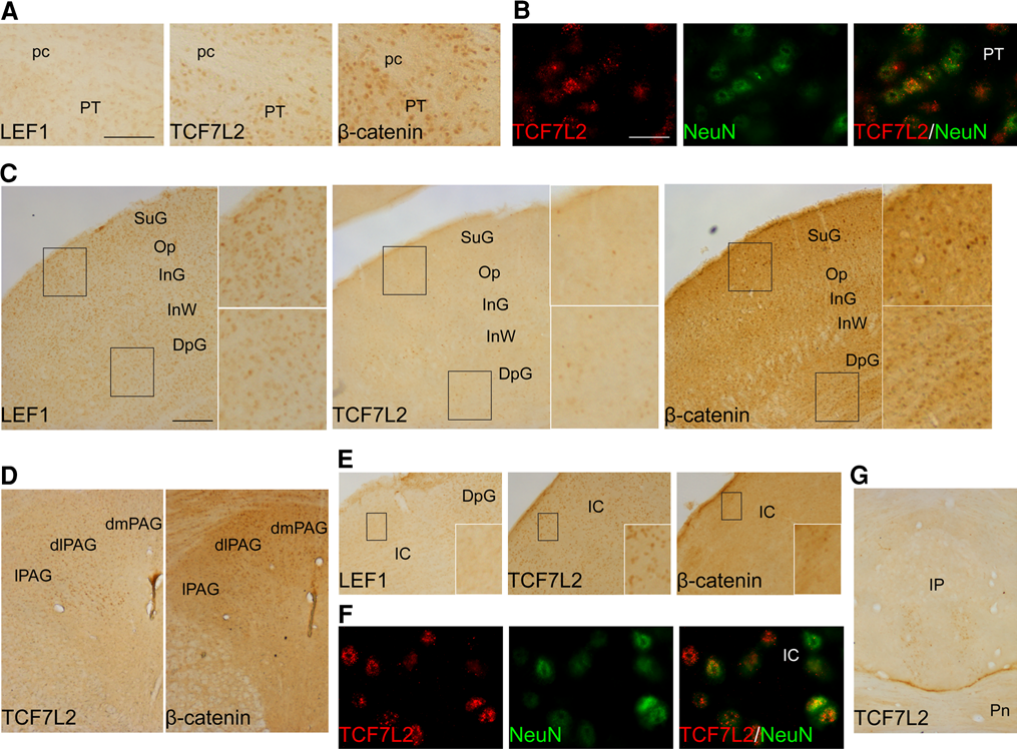
Distribution of LEF1, TCF7L2, and β-catenin proteins in the adult pretectum and midbrain. **a** The pretectal area exhibited TCF7L2- and β-catenin-positive cells, **b** which have a neuronal phenotype. **c** In the midbrain, LEF1 and nuclear β-catenin are expressed in all layers of the superior colliculus, whereas some TCF7L2-positive cells are present only in intermediate and deep layers. **d** The periaqueductal gray showed staining of both transcription factors and nuclear β-catenin in the lateral and dorsal parts. **e** Many TCF7L2-positive cells and some with nuclear β-catenin are present in the inferior colliculus, and **f** they exhibit a neuronal phenotype. **g** TCF7L2 is also expressed in the medial portion of the interpeduncular nucleus. *dlPAG* dorsolateral periaqueductal gray; *dmPAG* dorsomedial periaqueductal gray; *DpG* deep gray layers of the superior colliculus; *IC* inferior colliculus; *InG* intermediate gray layer of the superior colliculus; *InW* intermediate white layer of the superior colliculus; *IPN* interpeduncular nucleus; *lPAG* lateral periaqueductal gray; *Op* optic nerve layer of the superior colliculus; *pc* posterior commissure; *Pn* pontine nuclei; *PT* pretectal region; *SuG* superficial gray layer of the superior colliculus. *Scale bar* 100 μm for **a**. *Scale bar* 20 μm for **b** and **f**. *Scale bar* 200 μm for **c**–**e**

Cells in the pretectal region, including the large anterior pretectal nuclei (not shown), showed relatively moderate labeling for TCF7L2 with nuclear β-catenin but not LEF1 staining (Fig. 7a). Similar to the thalamus, TCF7L2-positive cells showed NeuN staining (Fig. 7b).

At the midbrain level TCF7L2, LEF1 and nuclear β-catenin were detected mainly in the superior colliculus (SC) and inferior colliculus (IC), and in the basal plate of the midbrain only few, scattered, TCF7L2-positive cells were observed (data not shown). In the SC, LEF1 and nuclear β-catenin were observed in all layers at a relatively moderate level (Fig. 7c). TCF7L2 was observed in the superficial layers but only in a scattered group of cells that showed labeling in the optic, intermediate, and deep layers (Fig. 7c). In the periaqueductal gray, LEF1 was detected only in the dorsomedial and dorsolateral parts (Fig. 7d), whereas TCF7L2 was seen in the dorsomedial, dorsolateral, and lateral parts. Nuclear β-catenin was observed in some groups of cells in the dorsal parts and to a lesser extent in the lateral part. At the level of the IC, we observed a high number of TCF7L2-positive cells compared with the SC, whereas LEF1 was not detected, and only few cells showed nuclear β-catenin staining (Fig. 7e). Combined immunofluorescence staining in the midbrain indicated that most of the TCF7L2-positive cells were neurons (Fig. 7f), but we also found some NeuN-negative cells in the IC, previously described to be Olig2-positive cells (Nazwar et al. 2009). Finally, in the rombencephalon, we detected TCF7L2 in the medial portion of the interpeduncular nucleus (Fig. 7g).

In summary, LEF1 and TCF7L2 proteins were observed in the epithalamus (only in the medial habenula), thalamus, and midbrain (both proteins in the SC but only TCF7L2 in the IC). The apparent presence of nuclear β-catenin in these structures implies the involvement of LEF1 and TCF7L2 proteins in the regulation of gene expression.

## Discussion

A growing body of evidence has implicated the effectors of canonical Wnt signaling, β-catenin and LEF1/TCF transcription factors, in mental and affective disorders. However, little is known about their actual physiological role in the adult brain. Here, we present in-depth study of LEF1/TCF mRNA and protein expression and isoform diversity in the adult mouse brain. We showed that TCF7L2 and LEF1 are highly expressed in the adult thalamus, with even higher levels at perinatal stages. We also showed that both transcription factors are expressed in other brain regions, including mainly the entorhinal cortex, ventral and medial pallidum, pretectum, superior and inferior colliculi, and periaqueductal gray. TCF7L2- and LEF1-positive cells were most often neurons, and the presence of TCF7L2 and/or LEF1 proteins correlated with the nuclear localization of β-catenin. The co-occurrence of the Wnt effectors in the specific subcortical structures specialized in the integration of diverse sources of sensory information suggests a role in coherent behavior, which is disrupted in some psychiatric disorders. Interestingly, both transcription factors underwent substantial isoform transitions during thalamic development, suggesting a developmental switch in gene expression regulation by LEF1/TCF and β-catenin.

### Expression of LEF1/TCFs in the developing and adult mouse cortex, thalamus, and midbrain

Wnt signaling has been shown to influence thalamic and midbrain patterning during embryonic development (McMahon and Bradley 1990; Thomas and Capecchi 1990; Pinson et al. 2000; Braun et al. 2003; Zhou et al. 2004; Hagemann and Scholpp 2012). The transcriptional output of the Wnt signaling pathway is mediated by the four LEF1/TCF paralogous effectors (Arce et al. 2006; Archbold et al. 2012). Only two of them, *Lef1* and *Tcf7l2*, are expressed in the developing mouse brain after E10.5 (Oosterwegel et al. 1993; Korinek et al. 1998b). The expression domain of *Tcf7l2* largely identifies the thalamus, pretectum, and alar part of the midbrain (SC and IC; with regional intensity differences), and *Lef1* is expressed in part of the telencephalon, thalamus, premammillary nucleus, and alar part of the midbrain (Oosterwegel et al. 1993; Cho and Dressler 1998; Jones and Rubenstein 2004; Bluske et al. 2009; Shimogori et al. 2010; Puelles et al. 2011). Previous in situ hybridization analyses showed that *Lef1* and *Tcf7l2* are still expressed in the adult mouse brain specifically in the thalamus, and *Lef1* is also expressed in the pretectum and alar midbrain (Jones and Rubenstein 2004; Shimogori et al. 2004; Lee et al. 2009). Our qPCR results are consistent with these studies and provide additional quantitative data, allowing comparisons of mRNA copy numbers among the members of the LEF1/TCF family. Although the general mRNA expression of *Tcf7* and *Tcf7l1* is very low in the forebrain and midbrain during late gestation and adulthood, *Tcf7l2* and *Lef1* exhibit substantial expression in these brain regions throughout development and adulthood. *Tcf7l2* is predominantly expressed in the embryonic thalamus and midbrain and practically not expressed in the cortex. *Lef1* exhibits moderate expression in the thalamus. The expression of *Tcf7l2* but not *Lef1* decreases postnatally, although its mRNA levels are still high. Therefore, the expression of *Tcf7l2* and *Lef1* in the thalamus and midbrain is maintained during adult stages, unlike many developmental transcription factors, such as *Dbx1*, *Dlx2*, *Gbx2*, *Olig3*, *Lhx1*, *Lhx2*, *Ngn1*, *Ngn2*, *Pax6*, and *Sox14* (Jones and Rubenstein 2004; Vue et al. 2007; Chen et al. 2009) as determined in the Allen Brain Atlas (http://mouse.brain-map.org; accessed 12 June 2012). This might suggest that these transcription factors are “terminal selectors” of these brain structures (Flames and Hobert 2009; Liu et al. 2010; Eade et al. 2012; Kratsios et al. 2012), i.e., their activity is required not only during development but also for the proper maturation and function of the thalamus and midbrain in adulthood. This is also supported by recent results indicating that thalamic identity continuously requires β-catenin signaling for its maintenance (Bluske et al. 2012).

### The adult brain expresses a unique combination of LEF1 and TCF7L2 isoforms

Our analysis revealed that the adult mouse brain expresses a distinct combination of LEF1/TCF protein isoforms compared with the embryonic brain or any other adult or developing tissue. First, both *Tcf7l2* and *Lef1* exhibit changes in isoform composition during brain development. The adult thalamus shows a predominance of full-length *Tcf7l2* isoforms, in contrast to developing stages during which the most prominent are the truncated isoforms that lack the β-catenin-binding domain and thus act as dominant-negative isoforms. In the case of *Lef1*, our analysis revealed a developmentally regulated increase in the inclusion of exon 6, which encodes a part of the domain involved in protein–protein interactions with different cofactors (Giese et al. 1995; Bruhn et al. 1997; Balmelle et al. 2004). An increase in exon 6 inclusion was also observed during thymus development and contributed to the activation of T cell antigen receptor α enhancer, the most critical checkpoint in T cell maturation (Carlsson et al. 1993; Mallory et al. 2011). Interestingly, during the first 3 postnatal weeks, the thalamus undergoes major morphological, neurochemical, and electrophysiological changes (Ramoa and McCormick 1994; De Biasi et al. 1997; Kidd and Isaac 1999; Parri and Crunelli 2002; Noutel et al. 2011); whether the developmental changes in the ratio between dominant-negative and full-length TCF7L2 isoforms and/or LEF1’s alternative splicing isoform are involved in these processes needs further investigation.

Second, the isoform composition of TCF7L2 in the brain is highly tissue specific compared with the broader expression of the other isoforms (Weise et al. 2010), present study). The main thalamic splice variant of TCF7L2 results from the coordinated exclusion of exon 4, sequence inclusion from the alternative 5′ splice site in exon 7, and exclusion from the alternative 3′ splice site of exon 9, which all encode parts of the “variable domain.” This region of the protein is located between the β-catenin and DNA-binding domains and has also been implicated in protein–protein interactions (Arce et al. 2006; Archbold et al. 2012). Furthermore, these alternative splicing events have been shown to influence transactivation activity and regulate the posttranslational modification of TCF7L2 (Pukrop et al. 2001; Weise et al. 2010).

Finally, the *C*-terminal part of TCFs exhibits complex alternative splicing regulation (Hovanes et al. 2000; Arce et al. 2006; Weise et al. 2010; Archbold et al. 2012). Interestingly, TCF7L2 expressed in the adult thalamus have a truncated C clamp, consistent with previous reports. The TCF7L2-S isoform was shown to be particularly abundant in the perinatal brain (Weise et al. 2010) and we demonstrated that it is also the predominant isoform in the adult thalamus and midbrain. The presence of a complete C clamp is indispensable for activation of genes implicated in proliferation (Hovanes et al. 2001; Hecht and Stemmler 2003; Atcha et al. 2007; Chang et al. 2008; Hoverter et al. 2012). In turn, the biological role of the TCF7L2-S isoforms is currently unknown. It has been shown that these isoforms bind DNA with lower efficiency, thus their transactivation capacity may rely more on protein–protein interactions than on DNA-binding specificity (Arce et al. 2006; Weise et al. 2010), allowing the regulation of different sets of genes in different molecular contexts (Mayall et al. 1997; Vadlamudi et al. 2005; Mahmoudi et al. 2010). We suppose that the lack of C clamp could provide molecular basis for neuron-specific regulation of gene expression by TCF7L2.

### Functional implication of differential patterns of LEF1 and TCF7L2 localization in the brain

The LEF1 and TCF7L2 proteins are particularly abundant in the thalamus and alar plate of the midbrain, and their expression domains often overlap, but their detailed localization shows some specific differences that may have functional implications given the specific functional diversification of the paralogs (Klingel et al. 2012). For example, in the thalamus—a central integrator of sensory information—TCF7L2 is prominent in every nuclei, whereas the LEF1 levels is relatively low or very low in some midline and intralaminar nuclei that provide projections to both the cortex and striatum (Sherman and Guillery 2002; Van der Werf et al. 2002; Basso et al. 2005; Jones 2009). The relative level of LEF1 is higher in the nuclei classified as first-order relays, which are implicated in the transmission and modification of sensory, auditory, and visual information to the cortex and are not connected with the striatum (Steriade 1997; Sherman and Guillery 2002). In the epithalamus, a node that links the forebrain and midbrain regions involved in regulating emotional behavior (Bianco and Wilson 2009; Hikosaka 2010; Aizawa et al. 2011), only the medial habenula exhibits the presence of both transcription factors. Interestingly, both divisions of the habenula have distinct functions and neuronal connectivity. The medial part projects almost exclusively to the interpeduncular nucleus (IP), where we found that it is one of the few nuclei in the rombencephalon that exhibit TCF7L2 expression. Moreover, according to a recent genoarchitectonic study in chickens (Lorente-Cánovas et al. 2012), the IP is subdivided into several portions. Our data suggest that these TCF7L2-positive cells are possibly part of the caudal centromedial subnucleus of the IP described in the avian study. In the superior colliculus, we also observed differences in the localization of LEF1 and TCF7L2 between functionally distinct subdivisions. LEF1 is apparently present in all layers, but TCF7L2 seems to be absent from the superficial gray and upper optic layers, which receive visual inputs from the retina and process visual information (Langer and Lund 1974). The deep layers are responsible for the integration of sensorimotor information (Krauzlis et al. 2004; May 2006; Cuppini et al. 2010) and reciprocally interconnected with the periaqueductal gray (PAG), which plays a critical role in various emotion-related, automatic, and reflex-like behaviors (Bandler and Shipley 1994; Floyd et al. 2000; Keay and Bandler 2001). Both the SC and PAG are also connected with thalamic nuclei (Krout and Loewy 2000; Krout et al. 2001).

Intriguingly, the regions that express LEF1 and/or TCF7L2 are involved in the integration of information from many sources and highly interconnected with each other. They are also implicated in many psychiatric disorders (Kang et al. 2008; Byne et al. 2009; Dommett et al. 2009; Cronenwett and Csernansky 2010; Hikosaka 2010; Pinault 2011; Fowler and Kenny 2012). The presence of LEF1 and TCF7L2 proteins in the thalamus and ventral midbrain correlates with nuclear β-catenin, suggesting that these transcription factors indeed regulate gene expression. For example, we previously showed that the LEF1/β-catenin complex in the thalamus regulates the expression of the *Cacna1g* gene, which encodes a voltage-gated T-type calcium channel (Wisniewska et al. 2010). Future studies will shed light on the target networks regulated by these transcription factors in the different brain structures and the extent to which the networks of LEF1 and TCF7L2 are functionally redundant.

## Conclusion

Our results showed that specific LEF/TCFs paralogs with a unique combination of isoforms are expressed in partially overlapping regions of the adult brain. Importantly, each paralog and isoform has been reported to have distinct properties, including specific protein–protein interactions with co-factors, different transactivation capabilities, and different target specificities. Therefore, the effects of gene expression regulation by LEF/TCFs are expected to be substantially different in the adult brain from those in non-neural tissues or in embryonic stages.
